# On the energetics and stability of a minimal fish school

**DOI:** 10.1371/journal.pone.0215265

**Published:** 2019-08-28

**Authors:** Gen Li, Dmitry Kolomenskiy, Hao Liu, Benjamin Thiria, Ramiro Godoy-Diana

**Affiliations:** 1 Japan Agency for Marine-Earth Science and Technology (JAMSTEC), Yokohama, Japan; 2 Graduate School of Engineering, Chiba University, Chiba, Japan; 3 Laboratoire de Physique et Mécanique des Milieux Hétérogènes (PMMH, UMR 7636), CNRS, ESPCI Paris–PSL Research University, Sorbonne Université, Université Paris Diderot, Paris, France; Coastal Carolina University, UNITED STATES

## Abstract

The physical basis for fish schooling is examined using three-dimensional numerical simulations of a pair of swimming fish, with kinematics and geometry obtained from experimental data. Energy expenditure and efficiency are evaluated using a cost of transport function, while the effect of schooling on the stability of each swimmer is examined by probing the lateral force and the lateral and longitudinal force fluctuations. We construct full maps of the aforementioned quantities as functions of the spatial pattern of the swimming fish pair and show that both energy expenditure and stability can be invoked as possible reasons for the swimming patterns and tail-beat synchronization observed in real fish. Our results suggest that high cost of transport zones should be avoided by the fish. Wake capture may be energetically unfavorable in the absence of kinematic adjustment. We hereby hypothesize that fish may restrain from wake capturing and, instead, adopt side-to-side configuration as a conservative strategy, when the conditions of wake energy harvesting are not satisfied. To maintain a stable school configuration, compromise between propulsive efficiency and stability, as well as between school members, ought to be considered.

## Introduction

The behaviors of living beings provide amazing examples of aggregated dynamics that result from complex social reasons [1–4]. Depending on the species, animals aggregate and modulate group cohesion to improve foraging and reproductive success, avoid predators or facilitate predation. Global cohesive decision and action for the whole group result from different types of interaction at the local scale. Fish schools, for instance, are an archetypal example of how local interactions lead to complex global decisions and motions [5]. Fish interact through vision but also by sensing the surrounding flow using their lateral line system [6]. From the fluid dynamics perspective, hydrodynamic interactions between neighbors have often been associated with swimming efficiency strategies, considering how each individual in the school is affected by the vortical flows produced by its neighbors. Breder [7] already recognized the importance of this issue, and more recent works have described how fish make use of vortices when swimming through an unsteady flow, whether produced by neighboring fish or by other features in the environment (see e.g. the review by Liao [8]). Concerning collaborative interactions between swimming fish, the first clear picture was proposed in the early 70’s by Weihs’ pioneering work [9]. He focused on interactions within a two-dimensional layer of a three-dimensional school, and proposed an idealized two-dimensional model in which each individual in the fish school places itself to benefit from the wakes generated by its two nearest neighbors, giving rise to a precise diamond-like pattern.

Weihs’ theory has been followed by extensive experimental verification generally comforting the idea of decreased energetic cost of locomotion in fish schools. Thus, Fields [10] reported decreased tail beat frequency as indicator to decreased swimming effort in groups of pacific mackerel (*Scomber japonicus*). Herskin and Steffensen [11] measured both tail beat frequency and oxygen consumption in sea bass *Dicentrarchus labrax*, and also found strong evidence for energy saving. Johansen et al. [12] estimated that trailing fish in a school (striped surfperch *Embiotoca lateralis*) benefited from over 25% reduction in oxygen consumption, based on correlations between swimming speeds, pectoral fin beat frequency, and oxygen consumption of solitary fish. Marras et al. [13] also inferred reduced costs of swimming from measurements of tail-beat frequency of grey mullet *Liza aurata* alone and in schools, combined with relationships between tail-beat frequency and activity metabolism. Interestingly, they found that all members of the school received energetic benefit regardless of their spatial position relative to neighbors. Halsey et al. [14] examined how water turbulence affected the tail beat frequency of sea bass *Dicentrarchus labrax* swimming in schools of different size. They reported a trend for attenuation of energy advantages which they explained by frequent short-term changes in fish position mediated by the turbulence. At the same time, they recognized that turbulence could modify the relationship between tail beat frequency and rate of oxygen consumption.

While consensus generally is maintained about energetic advantage of swimming in a group, the ubiquity of diamond formation as energy optimization policy has been subject to debate. Groups of red nose tetra fish *Hemigrammus bleheri* in shallow water, for instance, show strong preference for a phalanx configuration in high energy-demand swimming regimes [15]. Our understanding of the essential hydrodynamic interactions behind energy saving being insufficient to explain such behaviors observed in biological experiments, we resort in this work to the computational fluid dynamics (CFD) approach. Its most important advantage in the present context is that it provides a direct quantitative estimate to the hydrodynamic power in self-propelled swimming. Although the CFD modelling of collective swimming is not new, most of the prior work has been limited to groups of two-dimensional (2D) swimmers in 2D fluids [16–26].

We are only aware of two previous three-dimensional (3D) CFD studies of fish schooling. Large-eddy simulations by Daghooghi and Borazjani [27] modelled a large group of fish all swimming in the same plane as an infinite lattice of self-propelled in-phase synchronized swimmers. It was found that, for equal power, the fish in a rectangular formation with sufficiently small lateral distance swam 20% faster than alone. It was noticed, however, that the wake broke down into small, disorganized structures showing little evidence for constructive vortex interaction. As an alternative to the wake capture, channeling effect that enhances the flow velocity between swimmers was hypothesized to be the main energy-saving mechanism. A recent study by Verma et al. [28] included 2D and 3D numerical simulations. The 2D model was coupled with a deep reinforcement-learning algorithm to show that the collective energy savings in a fish school can be explained by a “smart” follower actively harvesting energy from the wake vortices behind its leader(s), achieving up to 32% increase in time-average swimming efficiency and 36% decrease in the cost of transport (*CoT*), with respect to a solitary swimmer. The control policy found in those 2D simulations was subsequently integrated within the 3D model in form of simplified rules. The 3D simulations showed 11% increase in efficiency and 5% decrease in *CoT*.

**Fig 1:**
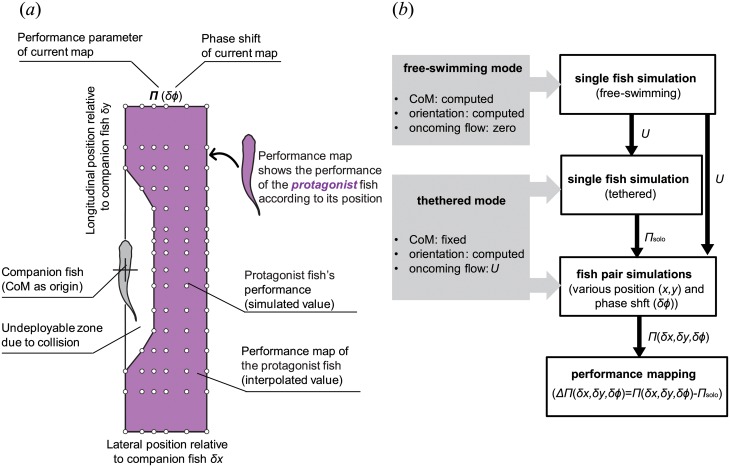
Numerical simulation methodology. (a) An explanation diagram of performance maps in Figs 3–7. Simulations were implemented by varying the relative longitudinal and lateral positions between two fish. To test the influence of phase difference, for each position (circles) we implemented four simulations (*δϕ* = 0, *T*/4, *T*/2 and 3*T*/4, respectively). Based on simulation results and interpolation, maps for swimming performance parameters were drawn. This performance map provide the performance value of the protagonist fish with its companion fish located at the origin. (b) (LHS) We conducted simulations in two modes: free-swimming (self-propelled) mode and tethered (fixed CoM) mode; (RHS) Procedure flow of simulations. Firstly, we simulated free-swimming single fish, obtained the terminal speed and apply to the rest simulations; We then simulated single fish swim and fish pair swim with CoM fixed. The performance of protagonist fish in the pair relative to the performance of a single fish is used to draw a performance map to demonstrate the influence of relative position and phase shift comprehensively. Here *U* is the terminal speed in single fish free-swimming, Π represents swimming performance parameter, such as net force, power, cost of transport, etc. Note that the same diagrams provide the performance of each of the two fish in the pair: the reader should select which of the two is considered as a protagonist, calculate its relative position and its phase shift with respect to its companion, and look for the corresponding point value on the performance map.

**Fig 2:**
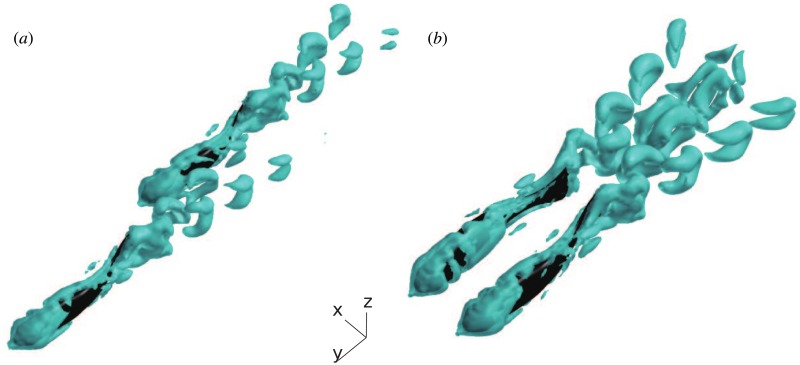
Three-dimensional flow visualization using an iso-surface of the Q-criterion [34]. (a) *δx* = 0.2*L*, *δy* = 1.25*L*, *δϕ* = 0; (b) *δx* = 0.5*L*, *δy* = 0, *δϕ* = *T*/2. For more examples of the wake topology, see [35].

The topic of our present study is local hydrodynamic interaction between individuals in small schools of tetra fish, as described in earlier experimental work by Ashraf et al. [15, 29]. A physical description of the local interactions between nearest neighbors, which are crucial in determining the whole group dynamics, still needs deeper insight. We therefore study the minimal subsystem of fish school, consisting in two fish swimming together, using a three-dimensional computational approach developed by Li et al. [30–32]. We investigate the consequences of spatial organization and kinematic synchronization on the energy expenditure of the two-fish school (see Fig 1) and the intensity of the pressure fluctuations induced by one individual on its neighbor. The fish are immersed in a sufficiently large numerical water channel (see Materials and methods). In the following description, we call ‘protagonist fish’ the one for which we report the swimming performance data such as forces, power, etc. The other one is called ‘companion fish’. We prescribe the temporal deformation of the fish midline having the same functional form for both fish, but with a phase shift *δϕ* (positive when protagonist lags behind the companion). It is known from past experiments [15, 29] that groups of tetra fish maintain some particular fixed configurations and constant gaits (see, e.g., Movie S1 in [33]). In our numerical study, we presume that all fixed configurations (i.e., fixed relative positions of the centers of mass (CoM) of the two fish) are realizable. We implement the simulations in order to clarify whether the observed configurations stand out in terms of favorable hydrodynamic interaction. Moreover, groups of tetra fish tend to align in one horizontal plane, i.e., the vertical offset between any two group members is smaller than each individual height [15, 29]. Considering that the hydrodynamic disturbances are the strongest in the same horizontal plane, we only investigate in-plane configurations in this work by imposing zero vertical separation between the two fish. The lateral spacing *δx* and the longitudinal spacing *δy* remain constant during each numerical simulation. Note that the protagonist is the follower and the companion fish is the leader if *δy* < 0, or vice versa if *δy* > 0. We perform a series of 312 simulations in total to realize parameter sweep in *δx*, *δy*. In addition, we test 4 different values of the phase shift *δϕ*. Fig 2 shows a visualization of the three-dimensional flow in two typical swimming configurations.

## Results

We conducted a simulation of a solitary fish in self-propelled swimming mode and obtained its terminal speed of 9.25 cm s^−1^ with a tail beat frequency of 8 Hz, which agrees well with the experiments [29]. We then applied an oncoming uniform flow at that velocity *U* = 9.25 cm s^−1^ (which gives a Reynolds number *Re* = 3700) and the same tail beat frequency of *f* = 8 Hz for all the rest of simulations in tethered mode. Note that the speed and the kinematics are not chosen arbitrarily, but representatively: a range of speeds of approximately 3 to 15 cm s^−1^ has been observed in the experiments (see Fig 2 in [29]), and 9.25 cm s^−1^ is almost in the middle. The experiments also suggested that fish had preferred combinations of frequency and amplitude depending on the speed. One of those is used in the simulations.

Thus, we obtained the swimming performance Π_*solo*_ of a solitary tethered fish and a performance map Π(*δx*, *δy*, *δϕ*) of the protagonist fish in pairwise simulations (see Fig 1), where the symbol Π represents a time-average performance parameter such as net force, power, etc. Among a variety of performance parameters, we chose the net longitudinal force *F*_∥_ and hydrodynamic power *P* as indicators of propulsive efficiency, and the lateral force *F*_⊥_, standard deviation of longitudinal force *s*.*d*.*F*_∥_, and standard deviation of lateral force *s*.*d*.*F*_⊥_ as indicators of stability. We quantify the effect of hydrodynamic interaction either as residual difference ΔΠ(*δx*, *δy*, *δϕ*) = Π(*δx*, *δy*, *δϕ*) − Π_*solo*_ or as percentage Π(*δx*, *δy*, *δϕ*)/Π_*solo*_ × 100%, whichever is more appropriate in its context.

### Effect on propulsive efficiency

#### Net longitudinal force

**Fig 3:**
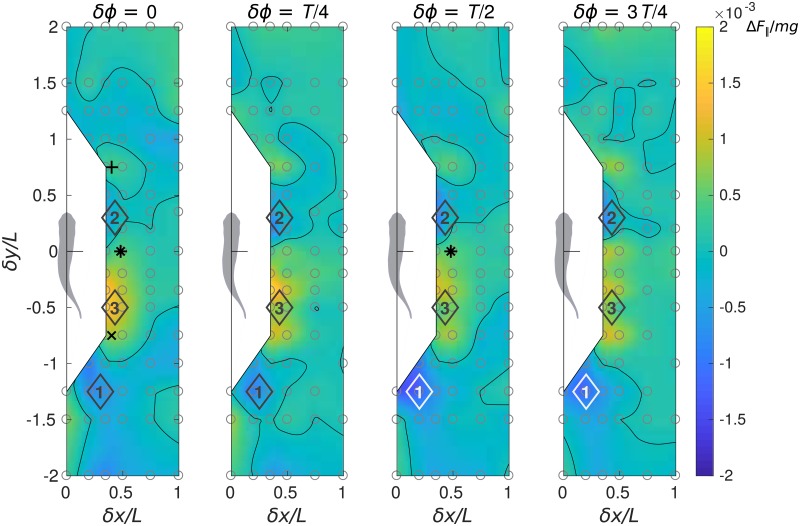
Performance maps of the protagonist fish in terms of normalized net longitudinal force. Normalized net longitudinal force is calculated as Δ*F*_∥_/*mg* = (*F*_∥_ − *F*_∥*solo*_)/*mg*.

The hydrodynamic interaction between the two fish induces an extra longitudinal force Δ*F*_∥_ on the protagonist fish, which is shown in Fig 3 in dimensionless form, normalized by the weight of the fish *mg*. The induced force Δ*F*_∥_ can act in the direction of drag or thrust, depending on the relative position of the two fish in the pair, and in magnitude it reaches 0.0018*mg*. Interestingly, Δ*F*_∥_ does not depend on *δϕ* as much as on *δx* and *δy*.

The protagonist fish experiences the largest drag when it ineptly plunges into the wake of its companion. This regime corresponds to the blue spots situated between *δy* = −2*L* and −*L*, see locations 1 in Fig 3, where *L* is the fish body length. Upon receiving such a penalty in Δ*F*_∥_, the protagonist fish in free swimming would adopt a more powerful stroke to maintain its speed and position, otherwise it would decelerate and fall behind.

A follower protagonist can experience positive Δ*F*_∥_ if placed in-line behind its leader companion, but this effect is confined to a narrow band (*δx* < 0.2*L*). The leader in this formation would experience positive Δ*F*_∥_ if *δϕ* = *T*/4 or 3*T*/4 and negative Δ*F*_∥_ if *δϕ* = 0 or *T*/2, but the magnitude of that force is negligible, i.e., there is no updraft. This finding contrasts with the strong upstream drafting observed in tandem arrangements of drag-generating flapping flags [36].

When the protagonist fish swims in a staggered side-by-side formation with its companion, in a slightly leading position (*δx* < 0.5*L* and 0 < *δy* < 0.6*L*, see locations 2 in Fig 3) it experiences slightly negative Δ*F*_∥_, while in a slightly trailing position (*δx* < 0.7*L* and −*L* < *δy* < 0, see locations 3 in Fig 3) the protagonist fish benefits from the largest positive Δ*F*_∥_. This implies that, in a staggered side-by-side formation with, e.g., *δy* ≈ ± 0.4*L*, extra propulsive force acting on the follower is accompanied by extra drag exerted on the leader. Therefore, in free swimming, this formation is likely to be unstable and to promote side-by-side arrangement with *δy* ≈ 0 (as in Fig 2b) so that the two fish equalize. Earlier experiments [29] using red nose tetra fish indeed showed that a pair of fish preferred phalanx formations with *δx* ≈ 0.6*L* and −0.2*L* < *δy* < 0.

Less compact staggered formations with *δx* ≈ 0.5*L* and *δy* ≈ ± 1*L* yield Δ*F*_∥_ ≈ 0 for both members. Therefore, such free-swimming formations can be sustainable. Incidentally, in [26], it was shown that two flexible self-propelled sheets in a two-dimensional flow maintained stable side-by-side formations with longitudinal separation less than 0.1*L*. Staggered formations were stable only for anti-phase swimmers with longitudinal separation greater than 1*L*. In [25], it was shown that two flexible self-propelled sheets can self-organize in an ‘alternate-leading’ state. The latter can be interpreted as a limit-cycle oscillation around a stationary point which is a precisely side-by-side formation. However, in those cases when initial lateral spacing was small enough, the stationary point switched to a staggered formation with longitudinal separation of approximately 1.3*L*. The longitudinal force augmentation/deficit patterns in Fig 3 are remarkably coherent with the stability results reported in [25, 26], in view of the differences in the numerical simulation setup.

**Fig 4:**
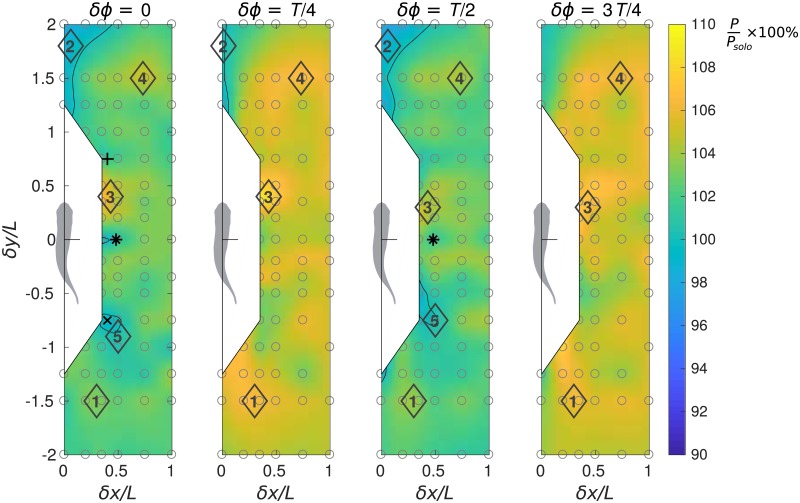
Performance maps of the protagonist fish in terms of relative power consumption. Relative power consumption is calculated as *P*/*P*_*solo*_ × 100%.

**Fig 5:**
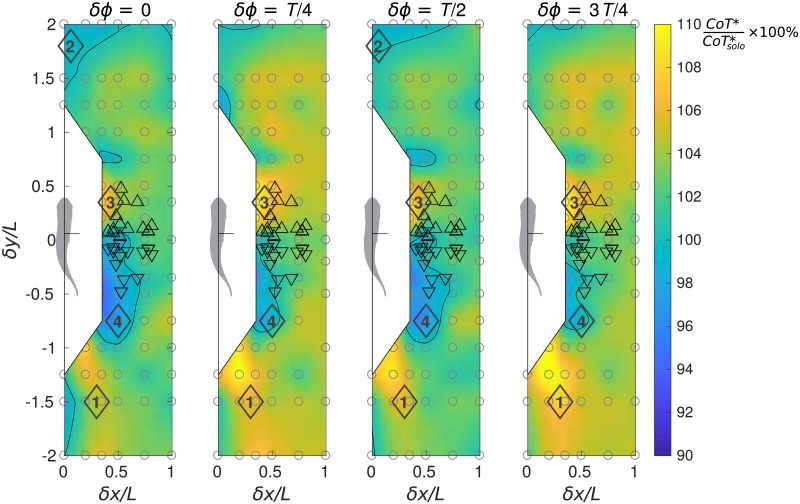
Performance maps of the protagonist fish in terms of cost of transport. Relative cost of transport is calculated as ${CoT}^{*}/{CoT}_{solo}^{*}\times 100\%$.

**Fig 6:**
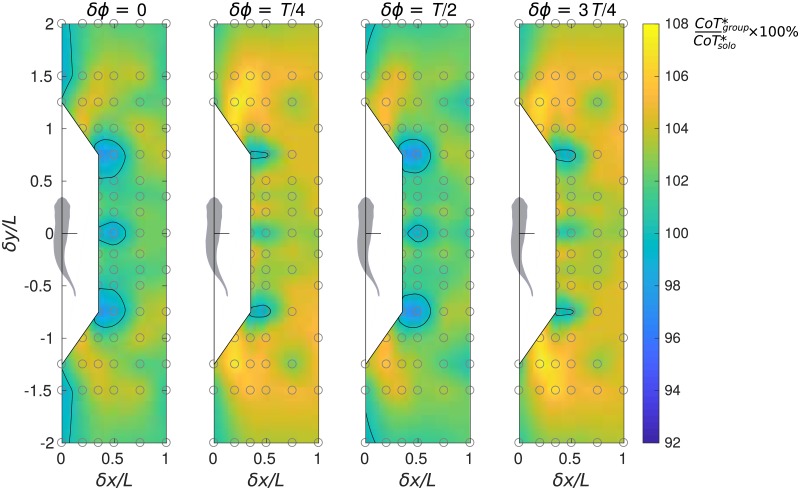
Performance maps of the average cost of transport of the group. Relative average cost of transport of the group is calculated as ${CoT}_{group}^{*}/{CoT}_{solo}^{*}\times 100\%$.

**Fig 7:**
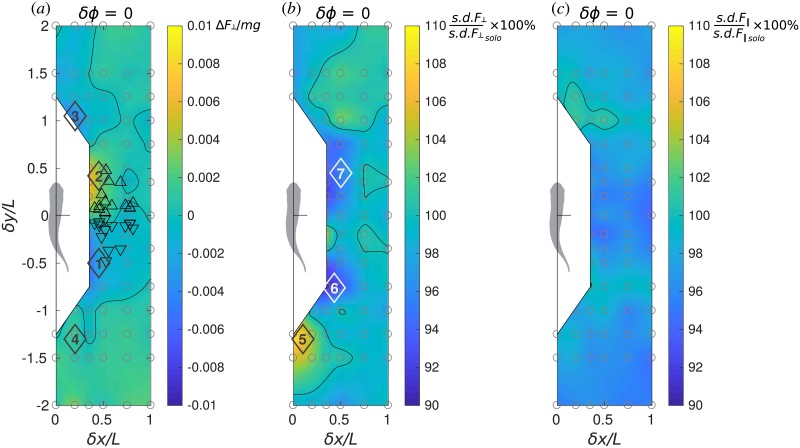
Stability performance maps. Performance maps of the protagonist fish in terms of (*a*) normalized lateral force, Δ*F*_⊥_/*mg*, (*b*) relative standard deviation of lateral force, *s*.*d*.*F*_⊥_/*s*.*d*.*F*_⊥*solo*_ × 100% and (*c*) relative standard deviation of longitudinal force, *s*.*d*.*F*_∥_/*s*.*d*.*F*_∥*solo*_ × 100%.

Note that the diagrams in Fig 3 render a spatial distribution of Δ*F*_∥_ applicable to either of the two fish in a pair. For example, if horizontal separation between the two fish is equal to 0.4*L* and vertical separation is 0.5*L*, the leader’s point is *δx* = 0.4*L*, *δy* = 0.5*L* and the respective follower’s point is *δx* = 0.4*L*, *δy* = −0.5*L*. If the two fish are synchronized in-phase (*δϕ* = 0), the first panel in Fig 3 gives Δ*F*_∥_ of both individuals. Similarly, in the case of anti-phase synchronization (*δϕ* = *T*/2), the third panel should be used. However, when the phase shift (lag) of the follower relative to the leader is equal to *δϕ* = *T*/4, one should evaluate the follower’s Δ*F*_∥_ using the second panel in Fig 3, but evaluate the leader’s Δ*F*_∥_ using the fourth panel, because the leader’s *δϕ* is of the opposite sign to the follower’s *δϕ*. Similarly, if the phase shift of the follower relative to the leader is equal to *δϕ* = 3*T*/4, one should refer to the fourth panel in Fig 3 for the follower and to the second panel for the leader. The performance maps shown below in Figs 4–7 also apply to both individuals.

#### Power

The hydrodynamic power consumption *P* of one fish in a pair varies with *δx*, *δy* and *δϕ* and it generally differs by less than 10% from *P*_*solo*_, see Fig 4. Remarkably, swimming with *δϕ* = 0 and *T*/2 is substantially less demanding in terms of power requirements than with *δϕ* = *T*/4 or 3*T*/4 (by up to approximately 5%). This may explain the preference for either in-phase or anti-phase synchronization observed in tetra fish in high energy demanding swimming regimes [29].

Relative spatial positioning also matters. When a follower protagonist fish is swimming in the jet behind its leader companion, in addition to the increased drag, it spends more power (locations 1, Fig 4, with *δx* > 0 and *δy* < −1). In this formation, the follower’s power consumption rises up to *P*/*P*_*solo*_ = 1.03 if the phasing is favorable (*δϕ* = 0) and to *P*/*P*_*solo*_ = 1.08 if the phasing is unfavorable (*δϕ* = *T*/4). Following the companion fish in a tandem formation with *δx* = 0 and *δy* < −1 is less penalizing for the protagonist. Positioning in tandem straight ahead of the companion may slightly lower the protagonist’s power consumption (locations 2, Fig 4).

Swimming on a diagonal in front of the companion fish may increase the power consumption (locations 3 and 4 in Fig 4), while swimming on a diagonal behind the companion (locations 5 in Fig 4) requires less power if *δϕ* = 0 or *T*/2. Staggered side-by-side formations are appealing when both the follower and the leader can enjoy extra thrust at negligible energetic cost. For instance, the case *δϕ* = 0, *δx* = 0.4*L* and *δy* = ∓0.75*L* (‘+’ and ‘×’ symbols in Figs 3 and 4) shows Δ*F*_∥_/*mg* = 0.0004, *P*/*P*_*solo*_ = 1 for the follower and Δ*F*_∥_/*mg* = 0.0007, *P*/*P*_*solo*_ = 1.01 for the leader, respectively. This means net benefit for the follower and possible benefit for the leader since, by a slight decrease in tail beat amplitude, thrust can be converted into power savings. However, swimming in this staggered formation requires good control skill from both individuals to keep the overall favorable relative position.

A side-by-side formation with *δx* = 0.48*L* and *δy* = 0 (‘*’ symbols in Figs 3 and 4) yields Δ*F*_∥_/*mg* = 0.0004, *P*/*P*_*solo*_ = 1 for both individuals if they are synchronized in-phase, and Δ*F*_∥_/*mg* = 0.0005, *P*/*P*_*solo*_ = 1.01 for both if they swim in anti-phase. This condition may be acceptable for the fish from the energetic point of view.

It should be reminded that the power data presented in this section assume no feedback control, no gait adaptation despite the force imbalance induced by hydrodynamic interaction. Therefore, the power should be analyzed in conjunction with the force. This is what we do in the next section.

#### A comprehensive result by *CoT* accounting for gait adjustment

Fish in schooling configurations need to adjust their gait to maintain their relative position. In our numerical simulations, we have prescribed the same tail beat frequency *f* and midline deformation envelope with amplitude *a* for both fish in the pair, see Methods. Real fish may adjust these parameters to reach the objective of steady swimming, but to remain synchronized, the group members must maintain equal frequency, while midline deformation can be used as a free control parameter.

The hydrodynamic interactions are weak enough to estimate the necessary adjustment of *a* using linear extrapolation. We therefore carry out an additional solitary fish simulation with *a* increased by 5%, i.e, $a_{solo}^{+}=1.05a_{solo}$, where the subscript ‘*solo*’ stands for the solitary fish. We use ‘+’ when we refer to the results of this additional simulation, and no superscript for the original simulation. The derivative of the longitudinal force with respect to the amplitude and the derivative of the power with respect to the longitudinal force are approximated as, respectively,

$$\begin{array}{c} \frac{dF_{∥}}{da}=\frac{F_{∥solo}^{+}-F_{∥solo}}{a_{solo}^{+}-a_{solo}}\;\;\text{and}\;\;\frac{dP}{dF_{∥}}=\frac{P_{solo}^{+}-P_{solo}}{F_{∥solo}^{+}-F_{∥solo}} \end{array}$$ (1)

at *a* = *a*_*solo*_. The above derivatives are used for calculating the adjusted amplitude $a_{solo}^{*}$ and power $P_{solo}^{*}$ of the solitary fish that would correspond to steady swimming at the same prescribed velocity *U*,

$$\begin{array}{c} a_{solo}^{*}=a_{solo}+{\left(\frac{dF_{∥}}{da}\right)}^{-1}\left(-F_{∥solo}\right)\;\;\text{and}\;\;P_{solo}^{*}=P_{solo}+\frac{dP}{dF_{∥}}\left(-F_{∥solo}\right), \end{array}$$ (2)

by ensuring the longitudinal force be close to $F_{∥}^{*}=0$. Similarly, for all points on the two-fish school diagrams versus separation *δx* and *δy* between the fish, we calculate

$$\begin{array}{c} a^{*}=a+{\left(\frac{dF_{∥}}{da}\right)}^{-1}\left(-F_{∥}\right)\;\;\text{and}\;\;P^{*}=P+\frac{dP}{dF_{∥}}\left(-F_{∥}\right), \end{array}$$ (3)

In (3), the values of *a*, *P* and *F*_∥_ correspond to the simulation data for the protagonist fish in the pair, for which the diagram is made.

There exist several different criteria commonly used to evaluate energetic efficiency of self-propelled swimming [37]. In this study, we choose the cost of transport *CoT* for its intuitive physical interpretation as energy consumed per distance traveled, which after normalization by the body weight becomes equal to

$$\begin{array}{c} CoT=\frac{P}{mgU}. \end{array}$$ (4)

Note that a direct application of this formula to the results of our numerical simulations would be problematic, because (4) implies that the fish is in steady forward swimming, which is in contradiction to the non-zero net longitudinal force in the simulations (see Fig 3). This problem is solved by using extrapolation to estimate the power under zero-longitudinal-force condition, as explained above.

The values of P* and $P_{solo}^{*}$ determined from (3) and (2) all correspond to the same swimming speed *U*. The cost of transport is thus equal to P*/*mgU* and $P_{solo}^{*}/mgU$, respectively. Therefore, the energetic benefit for the second fish in a pair can be quantified using the *CoT* ratio

$$\begin{array}{c} \frac{{CoT}^{*}}{{CoT}_{solo}^{*}}\times 100\%=\frac{P^{*}}{P_{solo}^{*}}\times 100\%. \end{array}$$ (5)

It should be reminded that our estimate is based on a linear approximation, i.e., all quadratic and higher order terms $O(F_{∥}^{2})$ are neglected in (3). If we combine the two equations (3) to eliminate *F*_∥_, we see that approximation error for P* is of order *O*((a* − *a*)^2^). If a* differs from *a* by 10% (i.e., (a* − *a*)/*a* = 0.1), one can expect the approximation error for the *CoT* be no greater than 1% order of magnitude (i.e., 0.1^2^) if d^2^ *P*/d*a*^2^ is of the same order of magnitude as *P*/*a*^2^ or less. In addition, (3) only corrects for the longitudinal force, but the lateral force remains unbalanced. Finally, the estimate includes the numerical simulation error due to the limitation of fixed CoM, absence of control, etc.

As shown in Fig 5, when the phase difference between the two fish is *δϕ* = *T*/4 or 3*T*/4, for the protagonist fish, the estimated cost of transport is globally greater than that of the in-phase and antiphase (*δϕ* = 0 or *T*/2). For all phase shift conditions, when the protagonist fish is exposed to the wake (vortex street) of the companion fish (locations 1, Fig 5), greater cost of transport is incurred. If the protagonist fish is located ahead of the companion fish (locations 2, Fig 5), it may slightly decrease the cost of transport. However, in that condition, the companion fish is located in the wake of the protagonist fish and it may prefer to relocate. In a staggered side-by-side formation, if the protagonist fish is slightly in front (locations 3, Fig 5), strong negative interaction occurs. Contrarily, if the protagonist fish is slightly behind, it can receive energetic benefit (locations 4). Still, in this situation, the companion fish is located on the diagonal in front of the protagonist fish and experiences negative influence. The relative positions of the fish pairs studied experimentally by Ashraf et al. [29] are indicated by triangles in Fig 5. The fish appear to avoid the regions of strong variation in the cost of transport.

#### Average *CoT* of the group

When estimating the energetics of schooling swimmers, in addition to taking the individual (protagonist) standpoint as discussed above, it is important to determine whether the collective behavior can bring net benefit to the group. It is evident from Fig 5 that side-by-side formations bring equal benefit to both members, but tandem formations give more advantage to the leader and staggered formations give more advantage to the follower. The two members of the pair may equalize their energy spending by dynamically changing places when they swim over long distances, making the time-average *CoT* of either individual equal to the group average *CoT*, which is defined as

$$\begin{array}{c} {CoT}_{group}^{*}=\frac{1}{2}({CoT}_{protagonist}^{*}+{CoT}_{companion}^{*}). \end{array}$$ (6)

In (6), the protagonist cost of transport ${CoT}_{protagonist}^{*}$ is found immediately from the diagrams in Fig 5 as explained above for a given formation defined by *δx*, *δy* and *δϕ*. The companion cost of transport ${CoT}_{companion}^{*}$ can be determined from the same diagrams by considering the adjoint configuration with *δx*, −*δy* and 2*π* − *δϕ*. The output of (6) is visualized in Fig 6, which shows the ratio ${CoT}_{group}^{*}/{CoT}_{solo}^{*}\times 100\%$ as a function of the protagonist’s position *δx*, *δy* and phase shift *δϕ* with respect to its companion. Note that, by construction, the group average *CoT* maps for the cases *δϕ* = 0 and *T*/2 are top-bottom symmetric, and the map for the case *δϕ* = 3*T*/4 is a mirror reflection of the one for *δϕ* = *T*/4.

Fig 5 suggests that the majority of compact formations of two fish are disadvantageous in terms of ${CoT}_{group}^{*}$, but there exist three types of profitable formations. One is the staggered diagonal formation with *δx* ≈ 0.45*L* and *δy* ≈ ±0.75*L*. It can lower the group average *CoT* in the cases of in-phase, anti-phase synchronization and quarter-period phase shift alike. The group can receive a bonus of up to 3% ${CoT}_{group}^{*}$. The second beneficial is the side-by-side formation with *δx* ≈ 0.5*L*, *δϕ* = 0 or *T*/2 saving up to 2% ${CoT}_{group}^{*}$. Note that small longitudinal offset, |*δy*| < 0.1*L*, is permissible. The third type is a tandem formation. In the case of in-phase synchronization, it saves up to 2% ${CoT}_{group}^{*}$. Longitudinal separation |*δy*| can be as large as 2*L*, but lateral separation is confined to |*δx*| < 0.1*L*. For anti-phase tandem formations we only find less than 1% ${CoT}_{group}^{*}$ benefit, and no benefit quarter-period phase shift cases, albeit there are hints of possible positive interaction upon larger longitudinal separation increasing beyond the *δy* parameter range considered in our study.

### Effect on stability

To study the effect of schooling on the stability of the fish pair swimming pattern, we examine the lateral forces and the fluctuation (represented by the standard deviation) of lateral and longitudinal forces. These results for in-phase swimming are summarized in the diagrams in Fig 7.

#### Lateral force

Receiving unbalanced lateral force may break the stable configuration between the two fish, unless the fish spends more effort to adjust the unbalanced lateral force to maintain their relative position, but such effort may reduce the energetic efficiency. In our numerical simulations, the fish does not implement such adjustment, since we prescribe a bilaterally symmetric body deformation envelop, see Material and methods. Instead, the fish is free to rotate about its CoM. While the time-average body orientation remains precisely forward in solitary swimming, it becomes significantly deflected to the left or to the right as soon as the flow symmetry is broken by the presence of a companion fish. Therefore, *F*_⊥_ includes contributions from two hydrodynamic interaction effects: bilateral asymmetry in the surface pressure distribution and reorientation of the fish in the laboratory reference frame.

As shown in Fig 7a, there are several locations that could lead to dramatically unbalanced lateral force: when the two fish swim side by side, a slight trailing position (location 1) pulls the protagonist fish towards its companion, on the contrary, a slight leading position pushes it apart (location 2). The two fish in a side-by-side configuration may align themselves (*δy* ≈ 0) to keep away from the zones of strong unbalance, which seems to agree with the behaviors in the experiments [29] (triangles in Fig 7a). Also, leading (location 3) and trailing (location 4) positions may also produce lateral imbalance. The results shown in Fig 7a for in-phase swimming are representative of all synchronizations.

#### Fluctuation of force

Within one tail beat cycle, the force exerted on the fish body fluctuates quasi-periodically. The fluctuation of lateral and longitudinal forces may also affect the stability in fish swimming. Halsey et al. [14] notice that fish may not be able to maintain station relative to their neighbors when they swim in a turbulent water stream. It is logical to conjecture that the leader’s wake can have a similar impact on the followers even if the ambient flow is laminar. Here, we utilize the standard deviation of the lateral and the longitudinal forces to quantify the fluctuation. Fig 7b and 7c show, respectively, *s*.*d*.*F*_⊥_ and *s*.*d*.*F*_∥_ for the in-phase synchronized cases. When comparing between these two components, it is important to bear in mind that, for a solitary swimmer, the lateral force fluctuation is three times as strong as the longitudinal force fluctuation, i.e., *s*.*d*.*F*_⊥*solo*_/*mg* = 0.0046 while *s*.*d*.*F*_∥*solo*_/*mg* = 0.0016. Fig 7b and 7c only show how this fluctuation is amplified of attenuated due to hydrodynamic interaction between the two fish when they swim in a pair. Thus, *s*.*d*.*F*_⊥_ can differ from *s*.*d*.*F*_⊥*solo*_ by as mush as ±9%, while *s*.*d*.*F*_∥_ only differs from *s*.*d*.*F*_∥*solo*_ by between −6% and + 2%. These facts taken together, we conclude that fluctuation in the lateral direction is more likely to be a strong destabilizing factor. This situation also holds for *δϕ* = *T*/4, *T*/2 and 3*T*/4 (not shown). Considering the spatial structure of *s*.*d*.*F*_⊥_ when *δϕ* = 0 (Fig 7b), we notice that location 5 corresponds to strengthened fluctuation that the fish may avoid. The staggered side-by-side locations 6 and 7 may be chosen to attenuate the lateral fluctuation. It should be mentioned, however, that the spatial position of the peaks of *s*.*d*.*F*_⊥_ varies with *δϕ* (not shown).

## Discussion

Our results show that the spatial organization and the kinematic synchronization of a pair of swimming fish—the *minimal school*—have a clear effect on two crucial aspects of schooling: energy expenditure and fluctuation minimization. We have examined the effect of the hydrodynamic interaction between the two fish on several performance parameters by probing forces and consumed hydrodynamic power on a fish that we have called the protagonist fish, while placing it in different positions and with a kinematic phase shift with respect to its neighbor (the companion fish).

**Fig 8:**
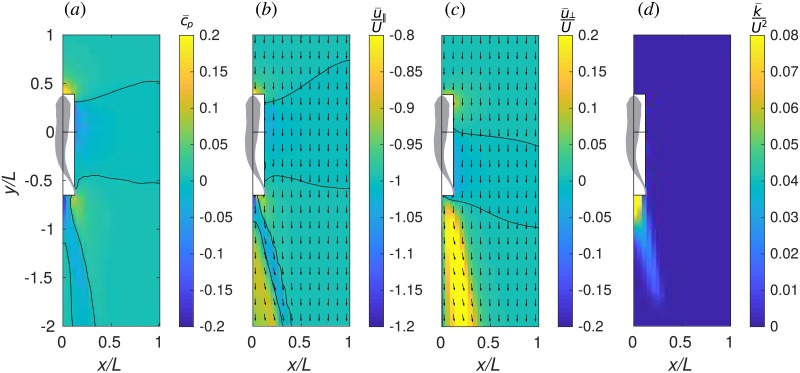
Time-average flow field near one fish. (a) pressure coefficient, ${\bar{c}}_{p}$, (b) longitudinal velocity, ${\bar{u}}_{∥}/U$, (c) lateral velocity, ${\bar{u}}_{⊥}/U$ and (*d*) fluctuation kinetic energy in the horizontal plane, $\bar{k}/U^{2}$. Vectors show the velocity in the horizontal plane.

The changes in hydrodynamics forces acting on the protagonist are in many cases driven by the induced time-average flow of the companion. The latter is displayed in Fig 8. Fig 8a shows the pressure coefficient ${\bar{c}}_{p}=2(\bar{p}-p_{\infty })/\rho U^{2}$. Overline denotes time averaging over a period of undulation, e.g., $\bar{p}=\frac{1}{T}\int_{0}^{T}pdt$. Fig 8b and 8c show, respectively, the longitudinal velocity ${\bar{u}}_{∥}$ and lateral velocity ${\bar{u}}_{⊥}$, normalized with the inflow velocity *U*. An in-plane velocity vector plot is superposed with each of the latter two color plots, for convenience. Fig 8d shows the energy of in-plane velocity fluctuation, $\bar{k}=\frac{1}{2}\bar{(u_{∥}-{\bar{u}}_{∥}{)}^{2}+(u_{⊥}-{\bar{u}}_{⊥}{)}^{2}}$, normalized by *U*^2^.

**Fig 9:**
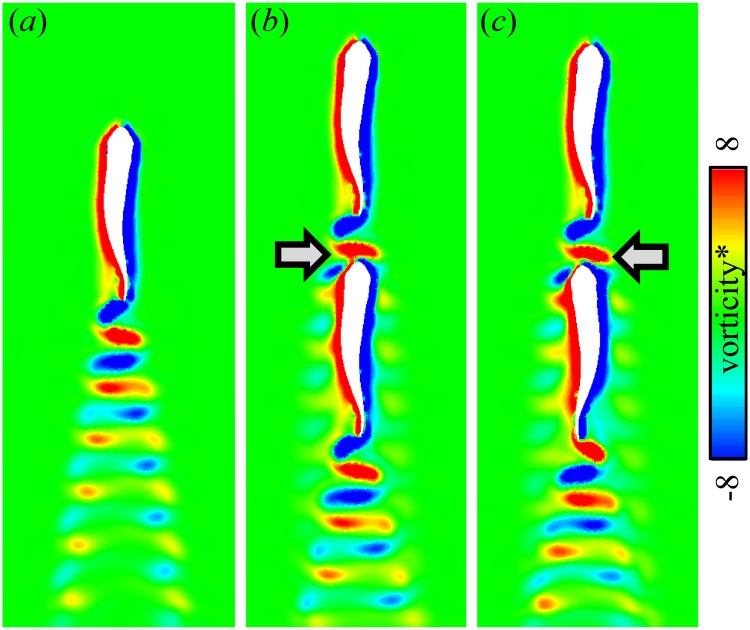
Interaction between the follower’s boundary layer vorticity with the leader’s wake. (a) Single fish; (b) Tandem formation with *δx* = 0, *δy* = ±1.25*L*, *δϕ* = 0; (c) Tandem formation with *δx* = 0, *δy* = ±1.25*L*, *δϕ* = *T*/2. Color plots show the vertical component of the dimensionless vorticity sampled on a horizontal plane. Instantaneous snapshots are shown such that the leader’s midline deformation is the same in all three cases. The arrows show the region of vortex capture. Note that the case (*b*) corresponds to Δ*F*_∥_ > 0 and (*c*) corresponds to Δ*F*_∥_ < 0.

Thus, the velocity plots unveil the complex structure of the jet behind the fish. In the right half-plane, which is visualized, the direction of the jet is backwards and to the right. Therefore, in the left half-plane, there is a symmetric jet directed backwards and to the left. These two jets correspond to the two rows of vortex rings in the wake behind a fish that can be seen in Fig 2. Between the jets, there is a narrow region where the water stagnates such that $|{\bar{u}}_{∥}|<U$. The fluctuation kinetic energy $\bar{k}$ is large in the jet, stressing the point that it is unsteady. A protagonist exposed to the companion’s jet will have to cope with an unsteady inflow velocity condition having the time average greater than *U*. Without gait adaptation, this implies additional drag on the protagonist. Since the jet is unsteady, varying *δϕ* can amplify or reduce this effect. If the protagonist finds itself fully immersed in the stagnation zone straight behind the companion, it swims in an effectively slower inflow, which implies additional thrust. However, when the follower swims very closely to the leader (*δy* = −1.25*L*), Δ*F*_∥_ may be either positive or negative, depending on the phase shift between the two fish. As shown in Fig 9, this may be related to the synchronization between the follower’s head and the leader’s tail. The follower receives positive thrust when it swings its head such as to capture the leader’s wake vortex on the left side of its body which has the same sign boundary layer vorticity (Fig 9b); otherwise, the vortex capture is destructive (Fig 9c). There exists a high pressure region in front of the fish and the velocity in that region is significantly smaller than *U*. This is the front stagnation point. The protagonist may benefit from swimming in the front stagnation region if the companion, but, in view of the small size of this region, the effect cannot be strong. Positioning straight ahead of the companion fish may slightly lower the power consumption (location 2, Fig 4). We hypothesize that, in a tandem configuration, the lateral velocity on the flow around the follower’s blunt head (as in Fig 8c) assists the lateral motion of the leader’s tail.

The hydrodynamic interaction mechanisms in side-by-side and staggered formations can be explained considering the time-average pressure field. The fish has a low pressure zone by its side. This zone is bounded by high pressure fringes in front and behind. A protagonist swimming side-by-side with its companion with *δy* = 0 is located in the middle of the low pressure zone therefore it does not experience any noticeable differential pressure from head to tail. If the protagonist leads by half of its body length, *δy* = 0.5*L*, its head is in the high pressure and its tail is in the low pressure zone, which sums up in a net backward force. If it falls behind by half of its body length, *δy* = −0.5*L*, its head is in the low pressure and its tail is in the high pressure, i.e., a net forward force is exerted on its body. There exist, of course, secondary effects due to nonlinear interaction of the flow fields induced by the two fish as well as unsteady interactions that depend on *δϕ*. Nevertheless, the features that are common for all *δϕ* in Fig 3 are perhaps the strongest, and they have their explanation from the time-average flow standpoint.

Regarding energy expenditure, we have used a cost of transport function (see Fig 5) that brings out two main conclusions. On the one hand, swimming in phase (*δϕ* = 0) or anti-phase (*δϕ* = *T*/2) is advantageous over the cases of quarter-period phase shift (*δϕ* = *T*/4 and 3*T*/4). Yet, it remains to be clarified whether the prevalence of in- and anti-phase lock behavior [29] stems from mechanical coupling akin to flagellar synchronization [38, 39] or from sensorimotor abilities. On the other hand, regardless of the phasing between neighbors, certain relative positions are beneficial or penalizing. Most notably, a side-by-side configuration with the protagonist fish slightly diagonally behind is beneficial for the protagonist fish, while lagging behind in the region of the wake of the companion fish is penalizing. When comparing the cost of transport maps with the positions of an experiment with a pair of tetra fish (triangles in Fig 5), high cost of transport zones appear to be avoided by the fish.

Considering the mechanisms such as updraft and channeling effect, as the number of fish involved in the collective behavior increases, the hydrodynamic benefit may accumulate as a quasi-steady linear interaction. These long-range interactions may be described analytically using dipolar far-field approximation [40]. Conversely, our results suggest that, as the number of fish decreases to two, unsteady and nonlinear interaction between the two fish becomes non negligible and specific flow structures and phase differences become important factors. It remains to be investigated how hydrodynamic influence evolves as the number of fish in a school increases.

Concerning the wake energy harvesting mechanism, our results suggest that, when a fish locates in the wake of the upfront leading fish, it becomes energetically inefficient. However, one should be aware that our conclusion is based on the tethered motion (fixed CoM) and absence of kinematic adjustment. A recent study by Verma et al. [28] shows that, when learning-based optimized kinematic adjustment is present, wake capture can be advantegeous. Therefore, the comparison between the present study and study of Verma et al. [28] demonstrates that there exists a distinction between wake capturing and wake energy harvesting: successful wake capture requires skills in sensing and adjustment, and if the fish (or an artificial swimmer) lacks those skills, wake capture may become energetically unfavorable. Besides the active mechanism, passive mechanisms based on structural compliance are also potential factors that may influence fish performance in school [26, 41].

We hypothesize that fish avoid wake capturing and adopt side-to-side configuration as a conservative strategy when energy harvesting is impractical due to adverse environmental conditions, physiological constraints, or other impeding factors. Furthermore, in comparison with two-dimensional wakes, three-dimensional fish wakes are geometrically more complex and less stable. The energy of vortex motion rapidly cascades to small-scale structures and dissipates, which hinders wake energy harvesting in 3D (cf. 36% decrement of *CoT* in 2D and only 5% decrement in 3D, in Verma et al. [28]). Further study is needed to quantify and fundamentally explain the difference between hydrodynamic interactions in the two-dimensional and the three-dimensional contexts.

The stability of the school has been studied examining the lateral forces and fluctuations of both lateral and longitudinal forces as functions of the relative position and kinematic phase shift. All phase lags produce qualitatively the same picture concerning lateral force and fluctuations, hence Fig 7 where only the *δϕ* = 0 case is shown. The fish would need to compromise between propulsive efficiency and stability, since the optimal positions for *CoT* and lateral force fluctuations do not coincide (for instance, compare between Figs 5a and 7a). Fish seeking for a stable position may suffer from high *CoT* and *vice versa*. In addition, fish may seek for mutually beneficial formations, since a schooling configuration exclusively beneficial to one member may be severely unfavorable to the other. A stable fish school configuration ought to be a concord between all members.

## Methods

**Fig 10:**
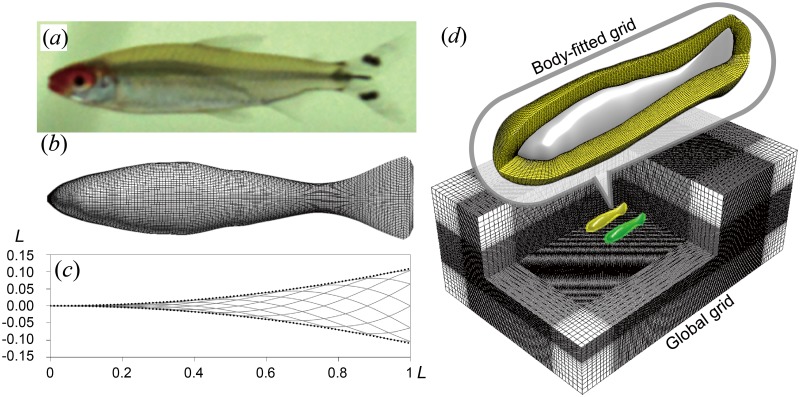
Computational fluid dynamics model. (a) Red nose tetra fish (*Hemigrammus bleheri*); (b) Surface model of Red nose tetra fish (dimension: 121 × 97); (c) A function (Eq 7) drives the instantaneous body shape. Variation of body length caused by this driving function was corrected to keep lateral excursion and body length constant at 1*L*. (d) Multi-blocked computational grid system consists of local fine-scale body-fitted grids (dimension: 121 × 97 × 20) plus a large stationary global grid (dimension: variant). Reprinted from [35] under a CC BY license, with permission from the Society of Aero Aqua Bio-mechanisms, original copyright 2019.

We developed an in-house three-dimensional overset grid numerical approach based on finite-volume method and programmed in FORTRAN 90 to simulate cyclic swimming of fish [30–32, 42]. The approach comprises surface models of the changing fish shape (dimension: 121 × 97), and local fine-scale body-fitted grids (dimension: 121 × 97 × 20) plus a large stationary global grid (dimension: various) to calculate the flow patterns around the fish with sufficient resolution (supportive information on grid resolution and size tests can be found in supplementary materials). As shown in Fig 10, to simulate a fish pair, two body-fitted grids were deployed, which deformed as the fish model deformed. The global grid surrounded the body-fitted grids and covered a sufficiently large domain to enclose the swimming fish and their wake. The ensemble of body-fitted grids and global grid was set up as a multi-blocked, overset-grid system based on a chimera grid scheme [43]. During the simulation, the body-fitted and global grids share values on their interfaces through inter-grid communication algorithm. The body was modelled on the silhouette of a Red nose tetra fish (*Hemigrammus bleheri*), with a body length of 4 cm, an average length measured in previous experimental study [29]. All cross-sections of the fish were modeled as ellipses. To reduce the complexity in modeling and computation, we assume that the hydrodynamic influence of all fins other than the tail fin is relatively minor, and neglect them in the model. Also, for the same reason, the gap of the fork-shaped tail fin is neglected, and the fish model has a triangle-shaped fin instead. The instantaneous body shape is driven by sinusoidal variation of the midline, cf. [32],

$$\begin{array}{c} H\left(l,t\right)=A\left(l\right)\sin(\frac{2\pi l}{\lambda }-2\pi ft) \end{array}$$ (7)

where *l* is the dimensionless distance from the snout along the longitudinal axis of the fish based on the length of the fish model *L*; *H*(*l*, *t*) is the dimensionless lateral excursion at time *t*;

$$\begin{array}{c} A\left(l\right)=a{\left(l/L\right)}^{2}, \end{array}$$ (8)

is the dimensionless amplitude envelope function at *l*; λ is the length of the body wave and it is set as 1.2*L*; *f* is the tail beat frequency defined as *f* = 8 Hz. We use *a* = 0.11 in all simulations, unless stated. These values of the model parameters are based on data from experiments [29]. Eq 7 may cause total body length along the midline to vary during the tail beat; this variation is corrected by a procedure that preserves the lateral excursion *H*(*l*, *t*) while ensuring that the body length remains constant. The correction algorithm is explained in S1 File. Procedure flow of simulations is shown in Fig 1b. We conducted simulations in two modes. In free-swimming (self-propelled) mode simulation, we simulated single fish swims in the horizontal plane with its center-of-mass (CoM) movements and body orientation determined by the hydrodynamic forces on the body, while oncoming flow was set as zero. By using free-swimming simulation, we obtained the terminal speed in single fish swimming and apply to the rest simulations. All the rest simulations were conducted in fixed CoM mode: we simulated a single fish or fish pair swimming with CoM and relative position fixed, while the rotational degree of freedom was still available to model the rotational recoil effect during swimming. This means that the fish can rotate if the torque exerted on it is not zero. Such semi-tethered condition is necessary for producing the performance maps. Otherwise, in free swimming with no feedback control, the fish may not necessarily hold formation.

The oncoming flow was set as the terminal speed obtained in free-swimming simulation. The Reynolds number of the simulations is defined as *Re* = *ρUL*/*μ*, where *ρ* is the water density, *U* is the swimming speed, *L* is the body length, and *μ* is the dynamic viscosity of water. The free-swimming simulation on a single fish rendered an equilibrium speed of 9.25 cm s^−1^. In all the rest simulations, the Re was set as 3700, and no turbulence model was applied in the simulation. Information on the validation of grid resolution, including the radial-direction grid resolution test, is provided in S1 File.

Because the two fish body-fitted grids were immersed in the global grid, interpolations between them defined the boundary conditions on their interface surface. On the fish body, non-slip condition was applied to set the flow velocity at the body surface equal to the local surface velocity. For the global grid, the upstream is set as the equilibrium speed 9.25 cm s^−1^ while pressure was set to zero, while at downstream and side (upper, lower, left and right) boundaries of the global grid a zero-gradient condition was enforced for both velocity and pressure.

The simulations on a fish pair were implemented by varying the relative longitudinal and lateral positions between two fish. The choice of using fixed CoMs for the two fish ensured that the relative position between the fish during swimming was unaffected by their complex interaction. Meanwhile, to test the influence of phase difference, for each position we implemented four simulations with varied phase shift between the two fish (*δϕ* = 0, *T*/4, *T*/2, 3*T*/4, respectively). Based on 312 of simulation results and interpolation among those results, we could construct the swimming performance map for different performance parameters. Fig 1a explains how to comprehend the performance maps (Figs 3–7). Note that a performance map is not a result of one simulation, but a summation of many simulations with a same phase shift between the two fish. Each circle in the map represents a simulated case, and the value at this point is the swimming performance (force, power, etc.) of the protagonist fish. The companion fish is placed at the origin point, while protagonist fish is assumed to be deployed in a range of relative position, which covers ±2*L* in longitudinal direction and from 0 to 1*L* in the lateral direction. Our definition of power is explained in S1 File. We calculate the time variation of the hydrodynamic power and apply time-averaging over one tail beat cycle.

## Supporting information

S1 File Electronic supplementary materials. (PDF)

## References

[1] Czirók A, Vicsek M, Vicsek T. Collective motion of organisms in three dimensions. Physica A: Statistical Mechanics and its Applications. 1999;264(1):299–304.

[2] Vicsek T, Zafeiris A. Collective motion. Physics Reports. 2012;517(3):71–140. 10.1016/j.physrep.2012.03.004

[3] Shaw E. Schooling fishes. Scientific American. 1978;66(2):166–175.

[4] Partridge BL. The structure and function of fish schools. Scientific American. 1982;246(6):114–123. 10.1038/scientificamerican0682-114 7201674

[5] Lopez U, Gautrais J, Couzin ID, Theraulaz G. From behavioural analyses to models of collective motion in fish schools. Interface Focus. 2012;2(6):693–707. 10.1098/rsfs.2012.0033 24312723 PMC3499128

[6] Partridge BL, Pitcher TJ. The sensory basis of fish schools: Relative roles of lateral line and vision. Journal of comparative physiology. 1980;135(4):315–325. 10.1007/BF00657647

[7] Breder CM. Vortices and fish schools. Zoologica: scientific contributions of the New York Zoological Society. 1965;50:97–114.

[8] Liao JC. A review of fish swimming mechanics and behaviour in altered flows. Philosophical Transactions of the Royal Society B: Biological Sciences. 2007;362(1487):1973–1993. 10.1098/rstb.2007.2082 PMC2442850 17472925

[9] Weihs D. Hydromechanics of Fish Schooling. Nature. 1973;241:290–291. 10.1038/241290a0

[10] Fields PA. Decreased swimming effort in groups of pacific mackerel (*Scomber japonicus*). American Zoologist. 1990;30(4):A134–A134.

[11] Herskin J, Steffensen JF. Energy savings in sea bass swimming in a school: measurements of tail beat frequency and oxygen consumption at different swimming speeds. Journal of Fish Biology. 1998;53(2):366–376. 10.1111/j.1095-8649.1998.tb00986.x

[12] Johansen JL, Vaknin R, Steffensen JF, Domenici P. Kinematics and energetic benefits of schooling in the labriform fish, striped surfperch *Embiotoca lateralis*. Marine Ecology Progress Series. 2010;420:221–229. 10.3354/meps08885

[13] Marras S, Killen SS, Lindström J, McKenzie DJ, Steffensen JF, Domenici P. Fish swimming in schools save energy regardless of their spatial position. Behavioral Ecology and Sociobiology. 2015;69(2):219–226. 10.1007/s00265-014-1834-4 25620833 PMC4293471

[14] Halsey LG, Wright S, Racz A, Metcalfe JD, Killen SS. How does school size affect tail beat frequency in turbulent water? Comparative Biochemistry and Physiology Part A: Molecular & Integrative Physiology. 2018;218:63–69. 10.1016/j.cbpa.2018.01.015 29408691

[15] Ashraf I, Bradshaw H, Ha TT, Halloy J, Godoy-Diana R, Thiria B. Simple phalanx pattern leads to energy saving in cohesive fish schooling. Proceedings of the National Academy of Sciences. 2017;114(36):9599–9604. 10.1073/pnas.1706503114 PMC5594674 28839092

[16] Bergmann M, Iollo A. Modeling and simulation of fish-like swimming. Journal of Computational Physics. 2011;230(2):329–348. 10.1016/j.jcp.2010.09.017

[17] Gazzola M, Chatelain P, van Rees WM, Koumoutsakos P. Simulations of single and multiple swimmers with non-divergence free deforming geometries. Journal of Computational Physics. 2011;230(19):7093–7114. 10.1016/j.jcp.2011.04.025

[18] Zhu X, He G, Zhang X. Flow-mediated interactions between two self-propelled flapping filaments in tandem configuration. Physical Review Letters. 2014;113:238105 10.1103/PhysRevLett.113.238105 25526164

[19] Gazzola M, Hejazialhosseini B, Koumoutsakos P. Reinforcement learning and wavelet adapted vortex methods for simulations of self-propelled swimmers. SIAM Journal on Scientific Computing. 2014;36(3):B622–B639. 10.1137/130943078

[20] Hemelrijk CK, Reid DAP, Hildenbrandt H, Padding JT. The increased efficiency of fish swimming in a school. Fish and Fisheries. 2015;16(3):511–521. 10.1111/faf.12072

[21] Becker AD, Masoud H, Newbolt JW, Shelley M, Ristroph L. Hydrodynamic schooling of flapping swimmers. Nature Communications. 2015;6:8514 10.1038/ncomms9514 26439509 PMC4600734

[22] Novati G, Verma S, Alexeev D, Rossinelli D, van Rees WM, Koumoutsakos P. Synchronisation through learning for two self-propelled swimmers. Bioinspiration & Biomimetics. 2017;12(3):036001 10.1088/1748-3190/aa6311 28355166

[23] Maertens AP, Gao A, Triantafyllou MS. Optimal undulatory swimming for a single fish-like body and for a pair of interacting swimmers. Journal of Fluid Mechanics. 2017;813:301–345. 10.1017/jfm.2016.845

[24] Park SG, Sung HJ. Hydrodynamics of flexible fins propelled in tandem, diagonal, triangular and diamond configurations. Journal of Fluid Mechanics. 2018;840:154–189. 10.1017/jfm.2018.64

[25] Peng ZR, Huang H, Lu XY. Collective locomotion of two closely spaced self-propelled flapping plates. Journal of Fluid Mechanics. 2018;849:1068–1095. 10.1017/jfm.2018.447

[26] Dai L, He G, Zhang X, Zhang X. Stable formations of self-propelled fish-like swimmers induced by hydrodynamic interactions. Journal of The Royal Society Interface. 2018;15(147). 10.1098/rsif.2018.0490 PMC6228486 30333246

[27] Daghooghi M, Borazjani I. The hydrodynamic advantages of synchronized swimming in a rectangular pattern. Bioinspiration & Biomimetics. 2015;10(5):056018 10.1088/1748-3190/10/5/056018 26447493

[28] Verma S, Novati G, Koumoutsakos P. Efficient collective swimming by harnessing vortices through deep reinforcement learning. Proceedings of the National Academy of Sciences. 2018;115(23):5849–5854. 10.1073/pnas.1800923115 PMC6003313 29784820

[29] Ashraf I, Godoy-Diana R, Halloy J, Collignon B, Thiria B. Synchronization and collective swimming patterns in fish (Hemigrammus bleheri). Journal of the Royal Society Interface. 2016;13(123). 10.1098/rsif.2016.0734 PMC5095228 27798281

[30] Li G, Müller UK, van Leeuwen JL, Liu H. Body dynamics and hydrodynamics of swimming fish larvae: a computational study. Journal of Experimental Biology. 2012;215(22):4015–4033. 10.1242/jeb.071837 23100489

[31] Li G, Müller UK, van Leeuwen JL, Liu H. Escape trajectories are deflected when fish larvae intercept their own C-start wake. Journal of The Royal Society Interface. 2014;11(101). 10.1098/rsif.2014.0848 PMC4223905 25401174

[32] Li G, Müller UK, van Leeuwen JL, Liu H. Fish larvae exploit edge vortices along their dorsal and ventral fin folds to propel themselves. Journal of The Royal Society Interface. 2016;13(116). 10.1098/rsif.2016.0068 PMC4843680 27009180

[33] Ashraf I, Godoy-Diana R, Halloy J, Collignon B, Thiria B. Supplementary material from “Synchronization and collective swimming patterns in fish Hemigrammus bleheri”; 2016. Available from: https://rs.figshare.com/collections/Supplementary_material_from_Synchronization_and_collective_swimming_patterns_in_fish_i_Hemigrammus_bleheri_i_/3500367/1. 10.1098/rsif.2016.0734 PMC5095228 27798281

[34] Hunt JCR, Wray AA, Moin P. Eddies, streams, and convergence zones in turbulent flows. In: Studying Turbulence Using Numerical Simulation Databases. vol. 2; 1988.

[35] Li G, Kolomenskiy D, Liu H, Thiria B, Godoy-Diana R. On the interference of vorticity and pressure fields of a minimal fish school. Journal of Aero Aqua Bio-mechanisms. 2019;8(1):27–33. 10.5226/jabmech.8.27

[36] Ristroph L, Zhang J. Anomalous hydrodynamic drafting of interacting flapping flags. Physical Review Letters. 2008;101:194502 10.1103/PhysRevLett.101.194502 19113271

[37] Li G, Liu H, Muller UK, Voesenek CJ, van Leeuwen JL. Hydrodynamic cost of transport determines optimisation strategy: larval fish provide insight into undulatory swimming. Submitted manuscript. 2019.

[38] Brumley DR, Wan KY, Polin M, Goldstein RE. Flagellar synchronization through direct hydrodynamic interactions. eLife. 2014;3:e02750 10.7554/eLife.02750 25073925 PMC4113993

[39] Kawamura Y, Tsubaki R. Phase reduction approach to elastohydrodynamic synchronization of beating flagella. Physical Review E. 2018;97:022212 10.1103/PhysRevE.97.022212 29548174

[40] Filella A, Nadal F, Sire C, Kanso E, Eloy C. Model of collective fish behavior with hydrodynamic interactions. Physical Review Letters. 2018;120:198101 10.1103/PhysRevLett.120.198101 29799263

[41] Ramananarivo S, Fang F, Oza A, Zhang J, Ristroph L. Flow interactions lead to orderly formations of flapping wings in forward flight. Physical Review Fluids. 2016;1:071201 10.1103/PhysRevFluids.1.071201

[42] Liu H. Integrated modeling of insect flight: from morphology, kinematics to aerodynamics. Journal of Computational Physics. 2009;228(2):439–459.

[43] Prewitt NC, Belk DM, Shyy W. Parallel computing of overset grids for aerodynamic problems with moving objects. Progress in Aerospace Sciences. 2000;36(2):117–172. 10.1016/S0376-0421(99)00013-5

