# Supplementary material for: On the energetics and stability of a minimal fish school
Source: PLoS One. 2019 Aug 28;14(8):e0215265. doi: 10.1371/journal.pone.0215265 (PMC6713342; doi:10.1371/journal.pone.0215265)
Supplement: S1 File — (PDF) [file pone.0215265.s001.pdf]

Electronic Supplementary Materials

On the energetics and stability of a minimal fish school

Gen Li[1,*], Dmitry Kolomenskiy[1,*], Hao Liu[2], Benjamin Thiria[3,+], and Ramiro Godoy-Diana[3,+]

[1]Japan Agency for Marine-Earth Science and Technology (JAMSTEC), Yokohama, 236-0001, Japan

[2]Chiba University, Graduate School of Engineering, Chiba, 263-8522, Japan

[3]Laboratoire de Physique et Mécanique des Milieux Hétérogènes (PMMH, UMR 7636), CNRS, ESPCI Paris--PSL Research University, Sorbonne Université, Université Paris Diderot, Paris, 75005, France

[*]Correspondence and requests for materials should be addressed to G. L. (email: ligen@jamstec.go.jp); D. K. (email: dkolom@jamstec.go.jp, dkolom@gmail.com)

[+]these authors contributed equally to this work

Part A Definitions of symbols, abbreviations and parameters 2

Part B Computational model information 3

Definition of power 5

Part C Computational results 7

Part A
Definitions of symbols, abbreviations and parameters

Table S1. Definitions of symbols and abbreviations

Symbol	Description
Π	Performance parameter
δx	Lateral spacing between the two fish
δy	Longitudinal spacing
$\delta\phi$ (positive: protagonist lags behind companion)	Phase difference
f	Tail beat frequency
T	Tail beat period
A	Dimensionless tail beat amplitude
U	Swimming speed
P	Power
$F_{\parallel}$ (positive: forward)	Net longitudinal force
$F_{\perp}$ (positive: push apart)	Net lateral force
CoT	Cost of transport
θ	Power per longitudinal force
l	Distance from the snout along the longitudinal axis
L	Length of the fish
λ	Length of the body wave
t	Time
H	Dimensionless lateral excursion of the midline
ρ	Water density
μ	Dynamic viscosity of water
Re	Reynolds number
$s.d.$	Standard deviation
CoM	Centre of mass
CFD	Computational fluid dynamics

Table S2. Reference values.

Parameter	Value
area of fish model (wetted area)	6.15×10^{-4} m^2
density of water ($\boldsymbol{\rho}_{\mathbf{w}}$)	1.00×10^3 kg•m^{-3}
mass of fish model (*m*)	6.31×10^{-4} kg
fish length (*L*)	4×10^{-2} m
volume of fish model	6.31×10^{-7} m^3
viscosity of water ($\boldsymbol{\mu}_{\mathbf{w}}$)	8.301×10^{-4} kg•m^{-1}•s^{-1}
$U_{\mathbf{solo}}$	9.25×10^{-2} m•s^{-1}
$P_{\mathbf{solo}}$	2.878×10^{-5} W

Part B
Computational model information

Table S3. Locations of the specific information regarding the computational approach.

specific information		where to find
methods	equations of hydrodynamic solution	Liu, 2009
	equations of motion solution	Li, et al., 2014, 2016 (in supplementary materials)
	coupling of hydrodynamic and motion solution	Li, et al., 2012, 2014, 2016
	multi-grid system and inter-grid communication	Liu, 2009; Li, et al., 2012
	body deformation control	Li, et al., 2012
	inter-body cell	Li, et al., 2016 (in supplementary materials)
validations	grid resolution independence test	Li, et al., 2014 (in supplementary materials)
	grid size independence test	Li, et al., 2016 (in supplementary materials)
	validation on hydrodynamic solution on oscillating cylinder, compared with experiment	Li, et al., 2014 (in supplementary materials)
	validation on hydrodynamic solution on fixed cylinder, compared with experiment	Li, et al., 2016 (in supplementary materials)
	validation on flow field on swimming fish, compared with PIV	Li, et al., 2012; Li, et al., 2016
	validation on motion solution on swimming fish, compared with experiment	Li, et al., 2012; Li, et al., 2014

Grid resolution

The grid resolution has been validated in Li, et al., 2014, at *Re*=6000. In this study, to prevent two adjacent fish body-fitted grids overlapping, we reduced the thickness of the body-fitted grid by applying fewer layers in the radial-direction, but the radial-direction grid resolution is still equivalent to that used in Li, et al., 2014. We implemented test on the radial-direction grid resolution to validate that the boundary layer on the fish was properly solved: in Fig. S1, black curve is the longitudinal force on fish computed with normal resolution in two randomly selected tail beat cycles, while red curve is that computed with doubled radial-direction grid resolution.

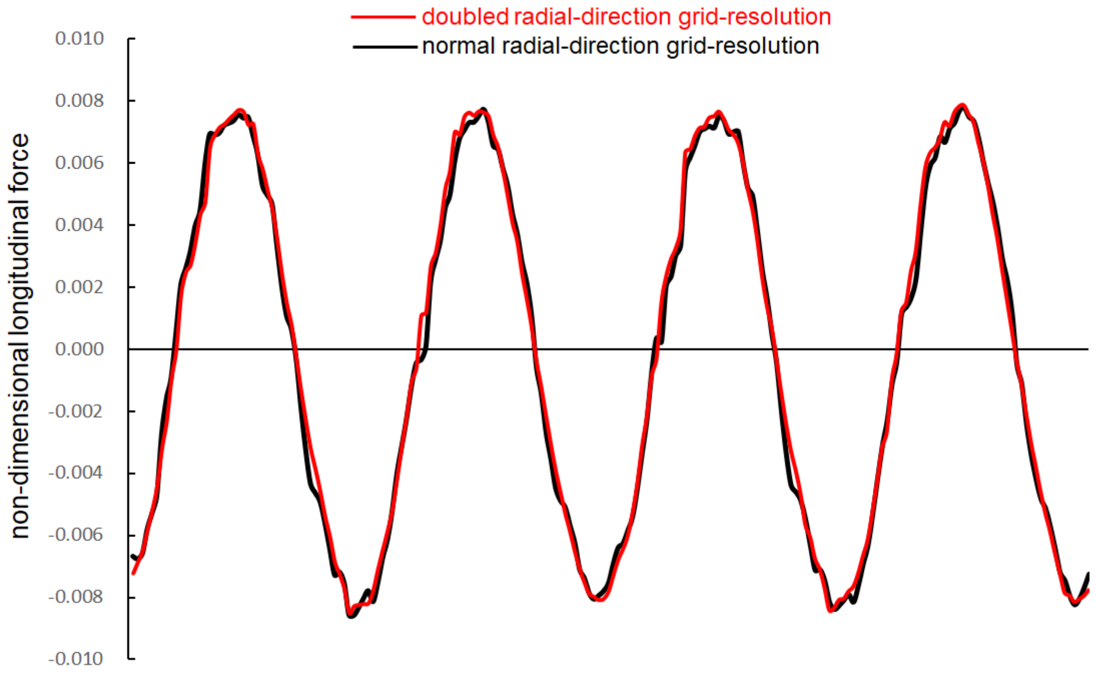

Fig.S1 Validation on the radial-direction grid resolution. Black curve: longitudinal force on fish computed with normal radial-direction resolution in two randomly selected tail beat cycles; red curve: longitudinal force on fish computed with doubled radial-direction grid resolution.

Body length correction algorithm

Since Eq. 7 may cause total body length along the midline to vary during the tail beat; this variation is corrected by a procedure that preserves the lateral excursion while ensuring that the body length remains constant. Fig. S2 explains the procedure of such correction: the deformed axis is longer than straight axis since when body joint i moves to position A by Δh, the length between joint i-1 and A is $\sqrt{\Delta L^2 + \Delta h^2} > \Delta L$. Hence, we let joint i move horizontally from position A to A* where the length between joint i-1 and A* is ΔL. Note that this correction procedure starts from the head, and each body joint

depends on its previous joint to confirm the position.

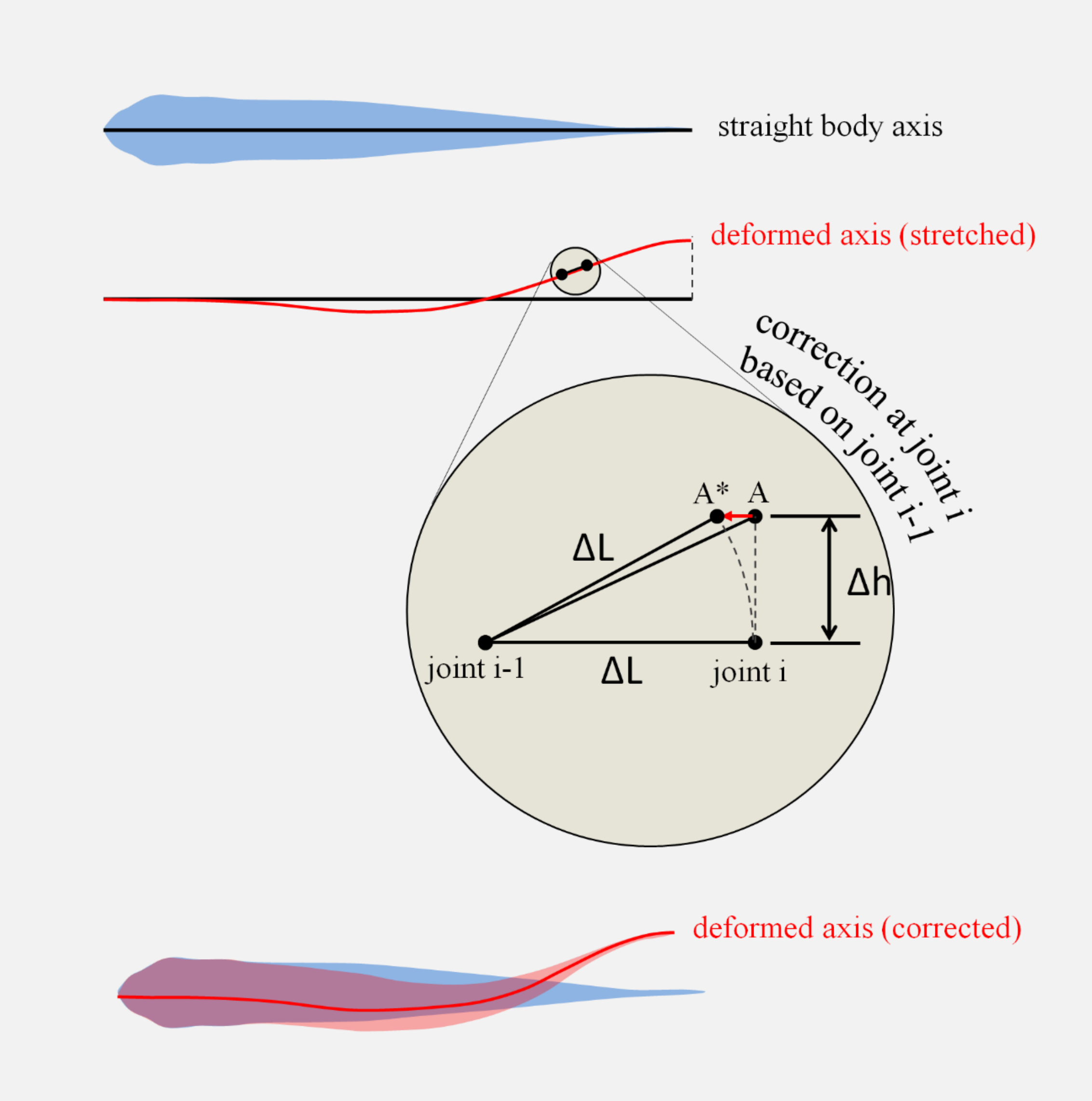

Fig.S2 body length variation caused by deformation is corrected by a procedure that preserves the lateral excursion while ensuring the body length constant.

Definition of power

Hydrodynamic power was calculated as the sum of the hydrodynamic work on the body surface, in a frame of reference that water is static, such that:

$$P_{hydro} = \sum_{surface} (\vec{\mathbf{F}}_{element} \cdot (\vec{\mathbf{U}}_{element} - \vec{\mathbf{U}}_{inflow}))$$

where P_{hydro} is the hydrodynamic power; $\vec{\mathbf{F}}_{element}$ is hydrodynamic force vector acting

on each surface element; $\vec{\mathbf{U}}_{element}$ is the velocity vector of this surface element in simulations; $\vec{\mathbf{U}}_{inflow}$ is the velocity vector of the inflow set only in tethered mode simulations.

References

[1] Liu, H. (2009). Integrated modeling of insect flight: from morphology, kinematics to aerodynamics. *Journal of Computational Physics*, *228*(2), 439-459.

[2] Li, G., Müller, U. K., van Leeuwen, J. L., & Liu, H. (2012). Body dynamics and hydrodynamics of swimming fish larvae: a computational study. *Journal of Experimental Biology*, *215*(22), 4015-4033.

[3] Li, G., Müller, U. K., van Leeuwen, J. L., & Liu, H. (2014). Escape trajectories are deflected when fish larvae intercept their own C-start wake. *Journal of The Royal Society Interface*, *11*(101), 20140848.

[4] Li, G., Müller, U. K., van Leeuwen, J. L., & Liu, H. (2016). Fish larvae exploit edge vortices along their dorsal and ventral fin folds to propel themselves. *Journal of The Royal Society Interface*, *13*(116), 20160068.

Part C
Computational results

Table S4. Computational results of the protagonist fish at $\delta\phi = 0$.

δx	δy	$\Delta F_{\parallel}(\mathrm{N})$	$\Delta F_{\perp}(\mathrm{N})$	$P(\mathrm{W})$	δx	δy	$\Delta F_{\parallel}(\mathrm{N})$	$\Delta F_{\perp}(\mathrm{N})$	$P(\mathrm{W})$
0	-2	2.19E-06	2.00E-06	2.935E-05	0.5	0	3.00E-06	1.01E-05	2.882E-05
0	-1.5	4.31E-06	1.39E-05	2.902E-05	0.5	0.2	3.23E-07	1.83E-05	3.008E-05
0	-1.25	2.92E-06	-3.01E-06	2.968E-05	0.5	0.35	-3.96E-07	1.74E-05	3.012E-05
0	1.25	-1.66E-06	-9.52E-06	2.846E-05	0.5	0.5	1.89E-07	1.74E-05	3.007E-05
0	1.5	-1.43E-06	-1.39E-05	2.833E-05	0.5	0.75	2.29E-06	7.13E-06	2.920E-05
0	2	-7.78E-07	4.40E-06	2.829E-05	0.5	1	-8.24E-07	-5.70E-06	2.968E-05
0.2	-2	-2.58E-06	2.33E-05	2.916E-05	0.5	1.25	-1.39E-06	-3.23E-06	2.955E-05
0.2	-1.5	4.08E-07	4.78E-06	2.977E-05	0.5	1.5	-6.50E-07	8.12E-06	2.987E-05
0.2	-1.25	-5.01E-06	1.22E-05	2.954E-05	0.5	2	9.66E-07	8.60E-06	2.892E-05
0.2	-1	-7.11E-06	7.50E-06	2.968E-05	0.75	-2	-1.76E-06	1.34E-05	2.930E-05
0.2	1	-1.26E-06	-3.37E-05	2.929E-05	0.75	-1.5	-1.41E-07	8.97E-06	2.979E-05
0.2	1.25	-7.23E-07	-3.42E-06	2.913E-05	0.75	-1.25	-1.58E-07	1.09E-05	2.897E-05
0.2	1.5	2.20E-06	-1.08E-05	2.952E-05	0.75	-1	-2.62E-06	4.17E-06	2.940E-05
0.2	2	-2.80E-07	-9.82E-06	2.828E-05	0.75	-0.75	-2.39E-07	-4.46E-06	2.976E-05
0.35	-2	-5.43E-06	4.64E-06	2.952E-05	0.75	-0.5	7.41E-07	-7.02E-06	2.925E-05
0.35	-1.5	-2.73E-06	1.06E-06	2.973E-05	0.75	-0.35	1.47E-06	-4.58E-06	2.929E-05
0.35	-1.25	-4.43E-06	-5.52E-06	2.942E-05	0.75	-0.2	8.01E-07	-4.11E-06	2.979E-05
0.35	-1	-4.48E-07	-5.98E-06	2.913E-05	0.75	0	8.41E-07	-7.33E-06	2.972E-05
0.35	-0.75	4.49E-06	-2.59E-05	2.859E-05	0.75	0.2	-2.98E-08	1.23E-06	2.973E-05
0.35	-0.5	1.06E-05	-3.83E-05	2.932E-05	0.75	0.35	-9.07E-08	-6.45E-06	2.954E-05
0.35	-0.35	1.02E-05	-3.00E-05	2.975E-05	0.75	0.5	-3.65E-07	8.13E-08	2.997E-05
0.35	-0.2	5.27E-06	-2.26E-05	2.947E-05	0.75	0.75	-4.22E-07	3.78E-06	2.955E-05
0.35	0	4.22E-07	1.36E-05	2.861E-05	0.75	1	-2.52E-06	1.80E-06	2.961E-05
0.35	0.2	-2.71E-06	3.99E-05	3.022E-05	0.75	1.25	-9.41E-07	3.33E-06	2.931E-05
0.35	0.35	-3.21E-06	4.25E-05	3.078E-05	0.75	1.5	1.26E-07	1.50E-07	3.034E-05
0.35	0.5	-2.50E-06	3.71E-05	3.060E-05	0.75	2	1.75E-06	1.14E-05	2.941E-05
0.35	0.75	2.40E-06	-4.46E-06	2.908E-05	1	-2	-1.58E-06	1.51E-05	2.945E-05
0.35	1	-2.16E-07	-6.35E-06	2.942E-05	1	-1.5	1.16E-06	8.14E-06	2.945E-05
0.35	1.25	-1.63E-06	-1.86E-06	2.953E-05	1	-1.25	7.71E-07	8.29E-07	3.000E-05
0.35	1.5	-2.88E-07	5.95E-07	2.989E-05	1	-1	-2.63E-06	6.76E-06	2.879E-05
0.35	2	1.32E-06	-7.91E-06	2.855E-05	1	-0.75	-2.72E-06	1.46E-06	2.985E-05
0.5	-2	-3.06E-06	3.18E-06	2.927E-05	1	-0.35	1.35E-06	-5.45E-06	2.965E-05
0.5	-1.5	-1.48E-06	1.01E-05	2.948E-05	1	0	4.36E-07	1.39E-06	2.970E-05
0.5	-1.25	-2.45E-06	6.29E-06	2.912E-05	1	0.35	7.88E-07	4.04E-06	2.971E-05
0.5	-1	-3.74E-07	8.20E-06	2.886E-05	1	0.75	-1.39E-06	-9.99E-07	2.926E-05
0.5	-0.75	4.63E-06	-1.29E-05	2.865E-05	1	1	-3.02E-06	-5.84E-06	2.910E-05
0.5	-0.5	6.69E-06	-9.24E-06	2.962E-05	1	1.25	2.52E-06	6.23E-06	2.974E-05
0.5	-0.35	5.76E-06	-7.50E-06	2.972E-05	1	1.5	2.65E-06	2.81E-06	2.974E-05
0.5	-0.2	4.79E-06	-7.14E-06	2.994E-05	1	2	2.02E-06	3.71E-06	2.922E-05

Table S5. Computational results of the protagonist fish at $\delta\phi = T/4$.

δx	δy	$\Delta F_{\parallel}(\mathrm{N})$	$\Delta F_{\perp}(\mathrm{N})$	$P(\mathrm{W})$	δx	δy	$\Delta F_{\parallel}(\mathrm{N})$	$\Delta F_{\perp}(\mathrm{N})$	$P(\mathrm{W})$
0	-2	2.41E-06	1.12E-05	2.986E-05	0.5	-0.5	3.89E-06	4.78E-06	2.973E-05
0	-1.5	5.39E-06	1.41E-05	3.027E-05	0.5	-0.35	7.23E-06	-6.67E-06	3.052E-05
0	-1.25	-1.42E-07	3.12E-06	3.056E-05	0.5	-0.2	2.65E-06	-2.44E-06	3.029E-05
0	1.25	2.82E-06	4.95E-06	2.821E-05	0.5	0	3.86E-06	9.72E-06	2.993E-05
0	1.5	-2.04E-06	-1.14E-05	2.868E-05	0.5	0.2	-2.51E-06	9.52E-06	3.015E-05
0	2	-3.02E-07	-1.17E-05	2.871E-05	0.5	0.35	1.39E-07	1.86E-05	3.100E-05
0.2	-2	-1.09E-07	2.24E-05	3.002E-05	0.5	0.5	-1.79E-06	5.22E-06	3.095E-05
0.2	-1.5	3.50E-07	7.58E-06	3.072E-05	0.5	0.75	4.21E-06	2.70E-06	3.018E-05
0.2	-1.25	-6.45E-06	2.16E-05	3.074E-05	0.5	1	-1.56E-06	-7.72E-07	3.047E-05
0.2	-1	-4.40E-06	2.00E-05	3.082E-05	0.5	1.25	1.08E-06	-5.93E-06	3.067E-05
0.2	1	1.38E-06	-3.59E-05	3.036E-05	0.5	1.5	2.67E-07	6.17E-06	3.057E-05
0.2	1.25	1.22E-06	-3.43E-06	3.014E-05	0.5	2	2.02E-06	4.35E-06	2.974E-05
0.2	1.5	4.72E-07	-1.18E-05	2.981E-05	0.75	-2	-1.30E-06	1.94E-05	2.994E-05
0.2	2	1.41E-06	-1.41E-05	2.906E-05	0.75	-1.5	-1.19E-07	1.44E-05	3.033E-05
0.35	-2	-4.43E-06	5.90E-06	3.015E-05	0.75	-1.25	8.93E-07	1.49E-05	2.958E-05
0.35	-1.5	-2.83E-07	3.81E-06	3.058E-05	0.75	-0.75	7.61E-07	-6.18E-06	3.055E-05
0.35	-1.25	-2.59E-06	-4.42E-06	3.040E-05	0.75	-0.5	-1.43E-07	-7.56E-06	3.004E-05
0.35	-1	1.20E-08	-1.63E-06	2.958E-05	0.75	-0.35	2.09E-06	-6.15E-06	2.992E-05
0.35	-0.75	6.38E-06	-2.66E-05	2.970E-05	0.75	-0.2	3.12E-06	-4.60E-06	3.068E-05
0.35	-0.5	4.59E-06	-1.61E-05	2.965E-05	0.75	0	1.12E-06	-2.66E-06	3.008E-05
0.35	-0.35	1.16E-05	-2.61E-05	3.061E-05	0.75	0.35	2.86E-07	-3.35E-06	3.019E-05
0.35	-0.2	6.44E-06	-3.36E-06	3.013E-05	0.75	0.75	-1.49E-06	2.30E-06	2.999E-05
0.35	0	1.61E-06	2.11E-05	2.940E-05	0.75	1.25	1.06E-06	7.11E-07	2.997E-05
0.35	0.2	-7.12E-06	2.33E-05	3.007E-05	0.75	1.5	8.44E-07	-1.54E-06	3.077E-05
0.35	0.35	-2.54E-06	3.52E-05	3.147E-05	0.75	2	1.94E-06	1.20E-05	3.001E-05
0.35	0.5	-2.89E-06	2.81E-05	3.095E-05	1	-2	-1.09E-06	1.28E-05	3.008E-05
0.35	0.75	4.03E-06	-2.55E-06	2.997E-05	1	-1.5	4.30E-07	6.09E-06	3.019E-05
0.35	1	1.21E-06	-8.47E-06	3.022E-05	1	-1	-9.50E-08	1.22E-06	3.029E-05
0.35	1.25	-8.80E-08	-2.53E-06	3.032E-05	1	-0.5	2.12E-07	-1.33E-06	3.027E-05
0.35	1.5	-7.01E-07	-4.63E-06	3.046E-05	1	-0.2	1.87E-06	-6.28E-07	3.054E-05
0.35	2	2.77E-06	-1.17E-05	2.938E-05	1	0	1.00E-06	2.69E-07	2.996E-05
0.5	-2	-2.78E-06	9.09E-06	3.009E-05	1	0.2	-6.29E-07	-1.01E-05	3.006E-05
0.5	-1.5	1.60E-06	1.04E-05	3.047E-05	1	0.5	4.18E-07	-3.10E-06	3.053E-05
0.5	-1.25	1.58E-07	6.09E-06	3.027E-05	1	1	8.49E-07	-8.28E-07	3.044E-05
0.5	-1	-9.25E-07	1.42E-05	3.002E-05	1	1.5	7.28E-07	6.27E-06	3.058E-05
0.5	-0.75	6.00E-06	-6.85E-06	2.966E-05	1	2	1.41E-06	5.61E-06	2.991E-05

Table S6. Computational results of the protagonist fish at $\delta\phi = T/2$.

δx	δy	$\Delta F_{\parallel}(\mathrm{N})$	$\Delta F_{\perp}(\mathrm{N})$	$P(\mathrm{W})$	δx	δy	$\Delta F_{\parallel}(\mathrm{N})$	$\Delta F_{\perp}(\mathrm{N})$	$P(\mathrm{W})$
0	-2	2.60E-06	7.12E-06	2.932E-05	0.5	0.2	4.49E-07	5.21E-06	2.988E-05
0	-1.5	1.75E-06	1.28E-05	2.941E-05	0.5	0.35	-7.51E-07	9.45E-06	3.005E-05
0	-1.25	-9.06E-06	7.74E-06	2.850E-05	0.5	0.5	-1.67E-06	1.10E-05	3.007E-05
0	1.25	-3.04E-06	-3.01E-06	2.805E-05	0.5	0.75	3.62E-06	4.41E-06	2.907E-05
0	1.5	-1.52E-06	-1.90E-05	2.861E-05	0.5	1	-6.65E-07	-4.43E-06	2.979E-05
0	2	-1.04E-06	1.12E-06	2.814E-05	0.5	1.25	-1.58E-06	2.88E-06	2.953E-05
0.2	-2	-1.28E-06	2.93E-05	2.953E-05	0.5	1.5	-3.00E-07	5.17E-06	2.989E-05
0.2	-1.5	-3.14E-06	1.40E-05	2.988E-05	0.5	2	1.49E-06	1.89E-06	2.891E-05
0.2	-1.25	-8.24E-06	2.59E-05	2.983E-05	0.75	-2	-2.18E-06	2.35E-05	2.908E-05
0.2	-1	-7.21E-06	2.46E-05	2.982E-05	0.75	-1.5	-1.61E-07	9.51E-06	2.975E-05
0.2	1	-1.24E-07	-2.94E-05	2.954E-05	0.75	-1.25	1.07E-07	1.14E-05	2.910E-05
0.2	1.25	-3.00E-07	-6.84E-06	2.917E-05	0.75	-1	-1.58E-06	1.03E-05	2.919E-05
0.2	1.5	-4.26E-07	-9.07E-06	2.931E-05	0.75	-0.75	-1.34E-06	-8.76E-06	2.985E-05
0.2	2	-6.62E-07	-5.48E-06	2.817E-05	0.75	-0.5	9.53E-07	9.33E-07	2.917E-05
0.2	-2	-3.10E-06	6.00E-06	2.937E-05	0.75	-0.35	9.90E-07	-3.48E-06	2.917E-05
0.35	-1.5	-2.60E-06	3.16E-06	2.993E-05	0.75	-0.2	1.16E-06	-5.74E-06	3.001E-05
0.35	-1.25	-3.89E-06	-1.26E-06	2.925E-05	0.75	0	1.32E-06	-3.54E-06	2.968E-05
0.35	-1	-4.64E-07	-6.36E-06	2.914E-05	0.75	0.2	2.40E-07	4.24E-06	2.989E-05
0.35	-0.75	3.88E-06	-3.31E-05	2.881E-05	0.75	0.35	-4.43E-07	-2.68E-06	2.950E-05
0.35	-0.5	2.27E-06	-7.90E-06	2.856E-05	0.75	0.5	-2.11E-07	2.13E-06	2.984E-05
0.35	-0.35	7.57E-06	-2.28E-05	2.880E-05	0.75	0.75	-7.88E-07	-6.90E-06	2.937E-05
0.35	-0.2	6.17E-06	2.06E-07	2.947E-05	0.75	1	-1.25E-06	5.06E-07	2.943E-05
0.35	0	2.64E-06	2.67E-05	2.968E-05	0.75	1.25	-8.19E-07	1.07E-06	2.928E-05
0.35	0.2	-6.73E-06	2.09E-05	3.029E-05	0.75	1.5	7.41E-07	-6.87E-06	3.013E-05
0.35	0.35	-6.05E-06	3.96E-05	2.999E-05	0.75	2	1.14E-06	1.28E-05	2.911E-05
0.35	0.5	-5.61E-06	2.15E-05	2.911E-05	1	-2	4.38E-07	1.16E-05	2.911E-05
0.35	0.75	2.38E-06	-5.53E-06	2.895E-05	1	-1.5	-1.58E-06	2.26E-06	2.937E-05
0.35	1	2.48E-07	-1.13E-05	2.963E-05	1	-1.25	1.40E-06	3.62E-06	2.955E-05
0.35	1.25	-1.88E-06	-4.38E-06	2.932E-05	1	-1	-2.59E-07	5.04E-06	2.930E-05
0.35	1.5	-5.20E-07	-2.37E-06	3.005E-05	1	-0.75	-1.57E-06	-4.58E-06	3.007E-05
0.35	2	9.92E-07	-9.46E-06	2.851E-05	1	-0.5	-1.34E-06	2.46E-06	2.935E-05
0.5	-2	-1.98E-06	9.30E-06	2.930E-05	1	-0.2	1.54E-06	-5.48E-06	3.003E-05
0.5	-1.5	-2.30E-07	1.27E-05	2.930E-05	1	0	5.39E-07	3.29E-06	2.922E-05
0.5	-1.25	-2.20E-06	1.27E-05	2.913E-05	1	0.2	1.86E-06	-7.59E-06	2.967E-05
0.5	-1	-7.41E-08	9.17E-06	2.908E-05	1	0.5	-4.09E-07	5.95E-06	2.965E-05
0.5	-0.75	5.66E-06	-4.31E-06	2.867E-05	1	0.75	-3.07E-07	-5.44E-07	2.926E-05
0.5	-0.5	3.73E-06	5.87E-06	2.899E-05	1	1	-2.13E-07	4.22E-06	2.947E-05
0.5	-0.35	5.78E-06	-9.54E-06	2.965E-05	1	1.25	2.90E-06	4.33E-06	2.938E-05
0.5	-0.2	3.67E-06	-1.43E-06	2.999E-05	1	1.5	-2.12E-07	-1.81E-06	2.924E-05
0.5	0	3.12E-06	1.34E-05	2.908E-05	1	2	-7.43E-07	4.22E-06	2.981E-05

Table S7. Computational results of the protagonist fish at $\delta\phi = 3T/4$.

δx	δy	$\Delta F_{\parallel}$(N)	$\Delta F_{\perp}$(N)	P(W)	δx	δy	$\Delta F_{\parallel}$(N)	$\Delta F_{\perp}$(N)	P(W)
0	-2	1.94E-06	8.85E-06	2.989E-05	0.5	-0.5	1.48E-06	-7.28E-07	2.958E-05
0	-1.5	-8.14E-07	1.89E-05	2.974E-05	0.5	-0.35	6.04E-06	-9.22E-06	3.013E-05
0	-1.25	-5.26E-06	-2.66E-06	2.911E-05	0.5	-0.2	2.95E-06	-1.42E-06	3.051E-05
0	1.25	4.19E-07	-9.25E-06	2.912E-05	0.5	0	4.77E-06	1.89E-06	3.022E-05
0	1.5	-1.86E-06	-1.75E-05	2.887E-05	0.5	0.2	-8.46E-08	1.37E-05	3.025E-05
0	2	9.36E-07	-8.88E-06	2.880E-05	0.5	0.35	-3.88E-07	1.82E-05	3.076E-05
0.2	-2	-1.51E-06	2.58E-05	3.016E-05	0.5	0.5	1.14E-06	7.66E-06	3.093E-05
0.2	-1.5	-2.27E-06	1.25E-05	3.032E-05	0.5	0.75	4.89E-06	-2.90E-07	3.006E-05
0.2	-1.25	-7.92E-06	1.78E-05	3.068E-05	0.5	1	-2.92E-07	-5.12E-06	3.045E-05
0.2	-1	-5.16E-06	1.06E-05	3.126E-05	0.5	1.25	-1.29E-06	1.13E-06	3.047E-05
0.2	1	2.28E-06	-3.16E-05	3.069E-05	0.5	1.5	2.34E-07	2.60E-06	3.064E-05
0.2	1.25	4.07E-07	-4.43E-06	3.002E-05	0.5	2	2.81E-06	3.92E-06	2.978E-05
0.2	1.5	-3.81E-07	-1.60E-05	2.975E-05	0.75	-2	-5.00E-07	1.65E-05	3.006E-05
0.2	2	9.82E-07	-8.79E-06	2.914E-05	0.75	-1.5	4.88E-07	9.52E-06	3.057E-05
0.2	-2	-2.93E-06	5.80E-06	3.008E-05	0.75	-1.25	5.25E-07	1.24E-05	2.979E-05
0.35	-1.5	-3.18E-06	6.92E-06	3.067E-05	0.75	-0.75	1.31E-06	-5.80E-06	3.044E-05
0.35	-1.25	-4.02E-06	-3.40E-06	3.051E-05	0.75	-0.35	1.06E-06	-6.76E-06	2.974E-05
0.35	-1	2.33E-07	1.56E-06	3.004E-05	0.75	0	1.38E-06	-7.12E-06	3.012E-05
0.35	-0.75	6.38E-06	-2.75E-05	2.996E-05	0.75	0.2	-1.02E-06	6.59E-06	3.017E-05
0.35	-0.5	4.52E-06	-1.43E-05	2.937E-05	0.75	0.35	8.12E-07	-2.35E-06	3.033E-05
0.35	-0.35	6.83E-06	-2.96E-05	2.941E-05	0.75	0.5	9.21E-07	-3.73E-06	3.071E-05
0.35	-0.2	3.89E-06	-1.51E-05	2.987E-05	0.75	0.75	-2.45E-07	-3.39E-06	3.012E-05
0.35	0	6.04E-06	7.97E-06	3.065E-05	0.75	1.25	1.04E-06	-1.29E-06	2.986E-05
0.35	0.2	-6.06E-06	3.84E-05	3.104E-05	0.75	1.5	-1.68E-07	1.85E-06	3.057E-05
0.35	0.35	-5.41E-06	4.85E-05	3.080E-05	0.75	2	2.36E-06	1.34E-05	3.013E-05
0.35	0.5	-4.45E-06	1.82E-05	3.037E-05	1	-2	-1.03E-06	8.43E-06	3.007E-05
0.35	0.75	4.52E-06	-4.29E-06	3.025E-05	1	-1.5	6.97E-07	3.48E-06	3.035E-05
0.35	1	-1.20E-07	-9.30E-06	3.020E-05	1	-1	3.35E-07	2.86E-06	3.030E-05
0.35	1.25	2.28E-06	-3.91E-06	3.058E-05	1	-0.5	3.96E-07	-1.35E-07	3.020E-05
0.35	1.5	-1.24E-07	2.19E-06	3.064E-05	1	-0.2	2.21E-06	-3.57E-06	3.068E-05
0.35	2	4.09E-06	-1.01E-05	2.953E-05	1	0	2.17E-06	6.41E-07	3.021E-05
0.5	-2	-7.96E-07	8.31E-06	3.004E-05	1	0.2	4.45E-07	-5.66E-06	2.973E-05
0.5	-1.5	1.73E-08	7.95E-06	3.029E-05	1	0.5	5.64E-07	1.89E-06	3.053E-05
0.5	-1.25	-2.06E-06	1.01E-05	3.006E-05	1	1	-4.23E-07	9.28E-08	3.063E-05
0.5	-1	5.29E-07	1.28E-05	3.023E-05	1	1.5	1.41E-07	-2.05E-06	3.066E-05
0.5	-0.75	6.42E-06	-6.89E-06	2.990E-05	1	2	1.89E-06	2.30E-06	3.003E-05
